# Precise 3D Lug Pose Detection Sensor for Automatic Robot Welding Using a Structured-Light Vision System

**DOI:** 10.3390/s90907550

**Published:** 2009-09-23

**Authors:** Jae Byung Park, Seung Hun Lee, Il Jae Lee

**Affiliations:** 1 Division of Electrical, Electronic and Computer Engineering, Chonbuk National University, Jeonju 561-756, Korea; E-mail: jbpark@chonbuk.ac.kr (J.B.P.); fallenstyner@gmail.com (S.H.L.); 2 Department of Mechanical Engineering, Chonbuk National University, Jeonju 561-756, Korea

**Keywords:** automatic robot welding, structured-light vision system, lug pose detection

## Abstract

In this study, we propose a precise 3D lug pose detection sensor for automatic robot welding of a lug to a huge steel plate used in shipbuilding, where the lug is a handle to carry the huge steel plate. The proposed sensor consists of a camera and four laser line diodes, and its design parameters are determined by analyzing its detectable range and resolution. For the lug pose acquisition, four laser lines are projected on both lug and plate, and the projected lines are detected by the camera. For robust detection of the projected lines against the illumination change, the vertical threshold, thinning, Hough transform and separated Hough transform algorithms are successively applied to the camera image. The lug pose acquisition is carried out by two stages: the top view alignment and the side view alignment. The top view alignment is to detect the coarse lug pose relatively far from the lug, and the side view alignment is to detect the fine lug pose close to the lug. After the top view alignment, the robot is controlled to move close to the side of the lug for the side view alignment. By this way, the precise 3D lug pose can be obtained. Finally, experiments with the sensor prototype are carried out to verify the feasibility and effectiveness of the proposed sensor.

## 1. Introduction

Automation of welding processes has been a challenging field of research in robotics, sensor technology, control systems and artificial intelligence because of its severe environmental conditions such as intense heat, fumes and so on [1]. In the field of robotics, industrial robot welding is by far the most popular application worldwide, since various manufacturing industries require welding operations in their assembly processes [2]. The most significant application of robot welding can be found in the automobile industry. In the case of the representative Korean automobile company, Hyundai Motor Company, the most manufacturing processes, except for delicate assembly processes, are automated with automotive assembly lines, and the welding process is almost fully automated. As a result, the productivity and quality of the products have been improved remarkably. On the contrary, the shipbuilding process is much less automated than the automobile manufacturing process due to its large-scale unstructured production environment. The welding process in shipbuilding is automated just 60%. Thus, the fact is that the study of robotic welding is still required in the field of shipbuilding, taking into consideration its complex and unstructured production environment.

**Figure 1.**
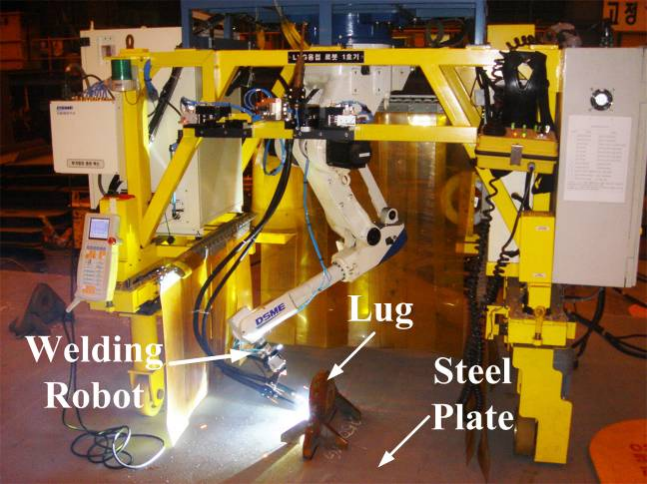
An automatic lug welding system with an overhead type robot manipulator developed by Daewoo Shipbuilding and Marine Engineering (DSME) Co., Ltd.

Shipbuilding is achieved by welding numerous steel plates according to a ship blueprint. Since the steel plates are too big and heavy to carry as is, a lug is attached to the plates as a handle, as shown in Figure 1. In this study, for robotic welding of the lug to the steel plate, a 3D lug pose detection sensor is proposed based on a structured-light vision system. In fact, a structured-light vision system has been commonly used for robotic welding with high precision and low disturbance [3,4]. In general, the structured-light vision system for robotic welding consists of a camera and more than one laser diode. In this case, the baseline (or distance) between a camera and a diode and the projection angle of the diode relative to the central axis of the camera determine the intrinsic system characteristics related to the performance. Kim *et al*. [5,6] proposed a mechanism to change the projection angle of a structured-light vision system according to the working distance. The system, however, needs additional parts such as an actuator and a controller, and also requires additional operating time to adjust the projection angle in accordance with the working distance. In this study, the proposed pose detection sensor consists of a camera and four laser line diodes. In our system, the baseline between a camera and a diode and the projection angle of a diode are key design parameters to determine the sensor performance. Thus, we first analyzed the sensor performance relative to the design parameters, and then determined the values for the parameters, taking the lug shape into consideration.

In robotic welding, the acquisition of the initial welding position is one of the most important steps [7,8]. In this study, we also focus on lug pose acquisition including its position and orientation through the coarse-to-fine alignment. First, the rough lug pose is obtained over the lug. According to the rough pose, the robot is controlled to move close to the side of the lug, and then the precise lug pose is obtained. Since the lug pose includes its position and orientation, the initial welding position and the welding line can be obtained from the lug pose. In this case, the structured laser lines are obtained by several image processing algorithms such as the vertical threshold algorithm [9], the Zhang-Suen thinning algorithm [10], the Hough transform algorithm [11] and the separated Hough transform algorithm robust to illumination change.

The organization of this paper is as follows. Section 2 describes the automatic robot welding procedure, and the design and performance analysis of the sensor. Section 3 proposes the coarse-to-fine alignment to obtain the precise lug pose consisting of position and orientation. In section 4, experimental results and discussion are provided to verify the feasibility and effectiveness of the proposed sensor. Finally, Section 5 will present concluding remarks.

## 2. Automatic Robot Welding System with a 3D Lug Pose Detection Sensor

### 2.1. Automatic Robot Welding Procedure

In this study, the automatic robot welding with the proposed 3D lug pose detection sensor proceeds in three stages: top view alignment, side view alignment and automatic welding stages. First, the lug pose consisting of both position and orientation is exactly obtained through the top and side view alignments. Next, the robot is controlled to move along the predefined welding path for automatic lug welding. In this study, we focus on the precise robot alignment with the proposed sensor since the success of the alignment is decisive in the success of the automatic robot welding.

**Figure 2.**
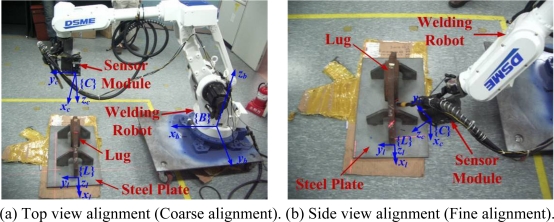
Possible robot configurations for the top and side view alignments.

Two possible configurations of the robot for the top and side view alignments are shown in Figure 2, where the proposed sensor is attached to the robot end-effector. In the figure, {*B*}, {*C*} and {*L*} represent the robot base frame, the camera frame and the lug frame, respectively. In this case, the problem to align the end-effector with the lug is the same problem to find {*L*} relative to {*C*}, where {*L*} relative to {*C*} can be simply transformed to {*L*} relative to {*B*} using the forward and inverse kinematics of the robot. Thus, the problem can be formulated as the 3D lug pose detection problem. In the top view alignment stage in Figure 2a, the lug frame {*L*} can be obtained relative to {*C*} with the premeasured lookup table (LUT) about the lug shape. However, the obtained frame {*L*} has some position and orientation errors since the camera resolution is relatively low at such a long distance. Thus, the top view alignment is called the coarse alignment. According to the obtained rough frame {*L*}, the robot is controlled to move close to the side of the lug for the side view alignment. In the side view alignment stage in Figure 2b, the fine alignment is carried out to find the precise lug frame {*L*}. Finally, according to the resultant lug frame {*L*}, the robot automatically welds. Table 1 describes the whole procedure of the automatic robot welding in details.

### 2.2. 3D Lug Pose Sensor Design

**Figure 3.**
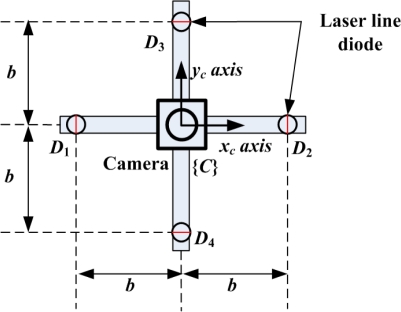
Front view of the 3D lug pose sensor with a monocular camera and four laser line diodes, *D*_1_, *D*_2_, *D*_3_ and *D*_4_.

The front view of the proposed 3D lug pose sensor, which consists of a camera and four laser line diodes, is shown in Figure 3, where *D_i_* for *i* = 1,2,3,4 indicates each diode, and *b* is the baseline (or distance) between the camera and each diode. The origin of the camera frame {*C*} coincides with the center position of the camera, and the *z_c_* axis of {*C*} is defined perpendicular to both *x_c_* axis and *y_c_* axis according to the right-hand rule. In this study, we employ FCB-EX480CP developed by Sony, Co. as a camera. The image size is 720 × 576 in pixel and the focal length *f* is 849 Pixels, where the focal length is empirically obtained by the MATLAB toolbox for camera calibration [12]. Also, we employ LM-6535MS developed by Lanics, Inc. as a laser diode, where the optical power is 20 mW, the wavelength is 658 nm, and the fan angle is 90°. The camera detects four laser lines projected on the lug put on the steel plate for obtaining the 3D lug pose.

**Figure 4.**
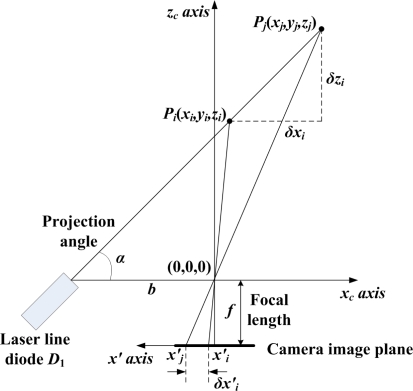
Acquisition of the 3D points, *P_i_*(*x_i_*,*y_i_*,*z_i_*) and *P_j_*(*x_j_*,*y_j_*,*z_j_*), on the laser line projected by the diode *D*_1_, and analysis of their resolutions.

The geometry of the camera and the laser diode *D*_1_ in the *x_c_-z_c_* plane [11] is shown in Figure 4. The 3D object point *P_i_*(*x_i_*,*y_i_*,*z_i_*) of the projected line of the diode *D*_1_ can be obtained relative to the camera frame {*C*} as follows:

(1) $$\left[x_{i}\;y_{i}\;z_{i}]=\frac{b}{f\;\text{cot}\;\alpha -x_{i}^{\prime }}[x_{i}^{\prime }\;y_{i}^{\prime }\;f\right]$$

where *α* is the projection angle which is defined as the angle between the central axis of *D*_1_ and the *x_c_* axis, and *p_i_*(*x′_i_*,*y′_i_*) is the measured point on the image plane. Similarly, for *p_j_*(*x′_j_*,*y′_j_*), the 3D object point *P_j_*(*x_j_*,*y_j_*,*z_j_*) can be obtained.

In this case, the baseline *b* and the projection angle *α* are the design parameters to determine the intrinsic sensor characteristics related to its performance. First, *b* is determined by the allowable sensor size to attach to the robot end-effector. In this study, *b* is set to be 7 cm. Next, for given *b*, *α* is determined according to the desirable sensor resolution and detectable range. Here, the sensor resolution is defined as the displacement in the 3D real space per one pixel in the image plane. Let *x′_i_* and *x′_j_* be the *i*th pixel and the (*i*+1)th pixel, respectively. Then, *δx′_i_* is one pixel since *δx′_i_* is (*x′_j_*−*x′_i_*). In this case, the displacements, *δx_i_* and *δz_i_*, for the *i*th pixel about the *x′* axis can be obtained by using Equation 1 as follows:

(2) $$\delta x_{i}=x_{i+1}-x_{i}=b\left(\frac{x_{i+1}^{\prime }}{f\;\text{cot}\;\alpha -x_{i+1}^{\prime }}-\frac{x_{i}^{\prime }}{f\;\text{cot}\;\alpha -x_{i}^{\prime }}\right)$$

(3) $$\delta z_{i}=z_{i+1}-z_{i}=\mathrm{bf}\left(\frac{1}{f\;\text{cot}\;\alpha -x_{i+1}^{\prime }}-\frac{1}{f\;\text{cot}\;\alpha -x_{i}^{\prime }}\right)$$

**Figure 5.**
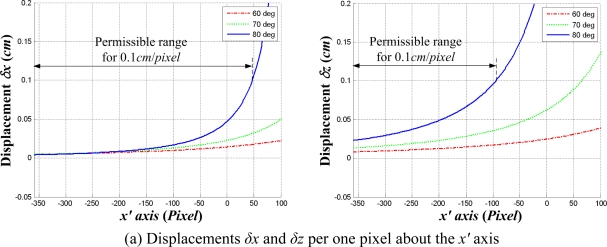

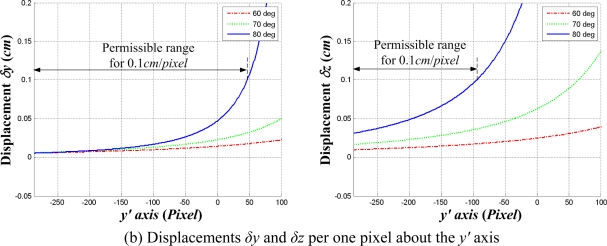
Permissible image ranges for the permissible resolution of 0.1 cm/pixel, where the baseline *b* is 7 cm.

Calculating Equations 2 and 3, the displacements *δx_i_* and *δz_i_* for *i* = −360, −359,…, 359 about the *x′* axis can be obtained for three projection angles of 60°, 70° and 80° as shown in Figure 5a. For the projection angle of 80° and the permissible resolution of 0.1 *cm*/*pixel* for fine alignment, the permissible image ranges are represented as an example. In other words, the robot must move close to the lug to satisfy the permissible range for automatic welding. In the coarse alignment, the permissible range is not satisfied since the robot is relatively far from the lug compared with the fine alignment. In this case, the resolution is exponentially reduced as shown in Figure 5. Thus, the fine alignment should be required for automatic robot welding. Similarly, the displacements *δy_j_* and *δz_j_* for *j* = −288, −287,…,287 about the *y′* axis and their permissible image ranges can be obtained as shown in Figure 5b. The Figure 5 shows that the permissible ranges decrease as the projection angle increases. Therefore, the projection angle *α* should be determined, taking all four resolutions in Figure 5 into consideration.

**Figure 6.**
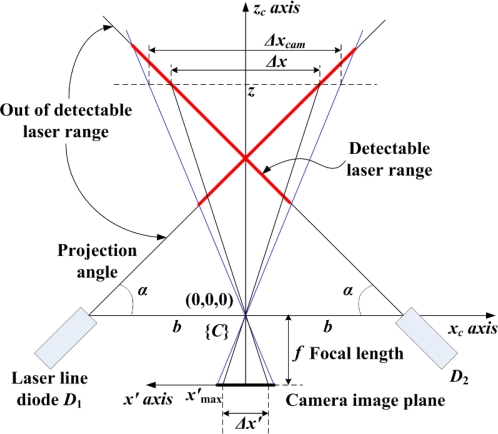
Detectable range about the *x_c_* axis according to the depth *z*.

To determine the projection angle *α*, the detectable range of the sensor should also be considered. The geometry of the camera along with two diodes, *D*_1_ and *D*_2_, in the *x_c_−z_c_* plane is shown in Figure 6, where two laser lines are symmetrically projected with the same *b* and *α*.

**Figure 7.**
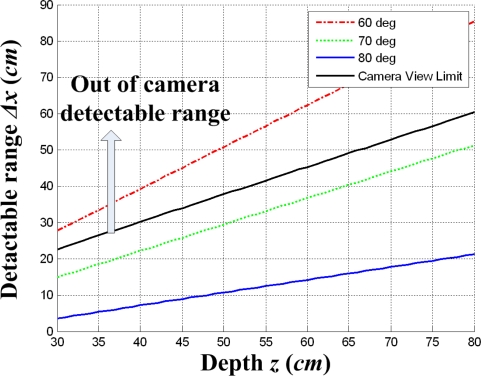
Detectable range *Δx* about the *x_c_* axis according to the depth *z*.

For given depth *z*, the detectable range *Δx* about the *x_c_* axis is obtained by using Equation 1 as follows:

(4) $$\Delta x=\frac{b\Delta x^{\prime }}{f\;\text{cot}\;\alpha -\frac{\Delta x^{\prime }}{2}}=2(z\;\text{cot}\;\alpha -b)$$

where *Δx′* is the width between two projected lines in image plane. The detectable range *Δx* increases as the depth *z* increases, and *Δx* decreases as the projection angle *α* increases as shown in Figure 7. In this case, the detectable range *Δx* should be bounded by the camera view limit *Δx_cam_*, where *Δx_cam_* is obtained as follows:

(5) $${\Delta x}_{\mathrm{cam}}={2x}_{\text{max}}^{\prime }\cdot \frac{z}{f}$$

where *x′*_max_ is 360 pixels, half the size of the image width. Thus, the projection angle of 60° is not allowable since the detectable range *Δx* for *α* = 60° is out of the camera detectable range *Δx_cam_* as shown in Figure 7. Similarly, the detectable range *Δy* about the *y_c_* axis can be obtained in the *y_c_*−*z_c_* plane, where the baseline is set as *b* but the projection angle is differently set as *β*. According to both detectable ranges, *Δx* and *Δy*, the projection angles *α* and *β* should be determined, where the detectable ranges are determined by the lug size. Through the above mentioned design process, we can determine proper projection angles *α* and *β*, taking a trade-off between the sensor resolution and the detectable range into consideration.

## 3. 3D Lug Pose Detection

### 3.1. Rough Lug Pose Detection

**Figure 8.**
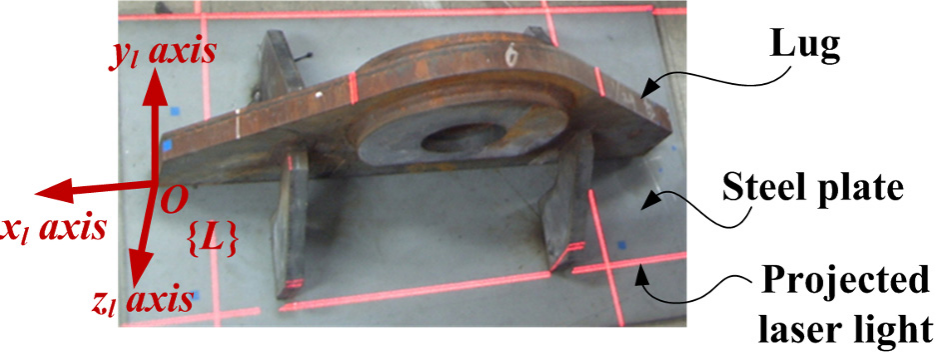
Top view image with the lug frame {*L*}.

The top view alignment is first carried out for detecting the rough lug pose. In this case, the lug pose detection is the same problem as the lug frame acquisition. The local frame {*L*} of the lug which is temporarily welded to the steel plate is defined as shown in Figure 8. The *x_l_* axis and *y_l_* axis of {*L*} are defined in the longitudinal and lateral directions of the lug, respectively. The *z_l_* axis can be obtained by the cross product of *x_l_* with *y_l_*. In this case, four laser lines are projected on the lug and the steel plate.

**Figure 9.**
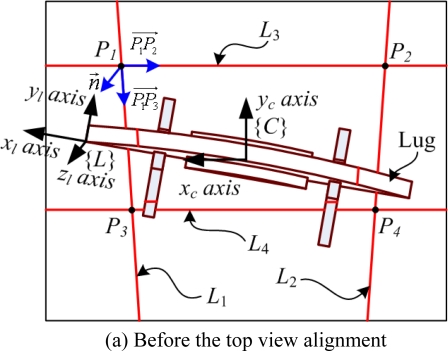

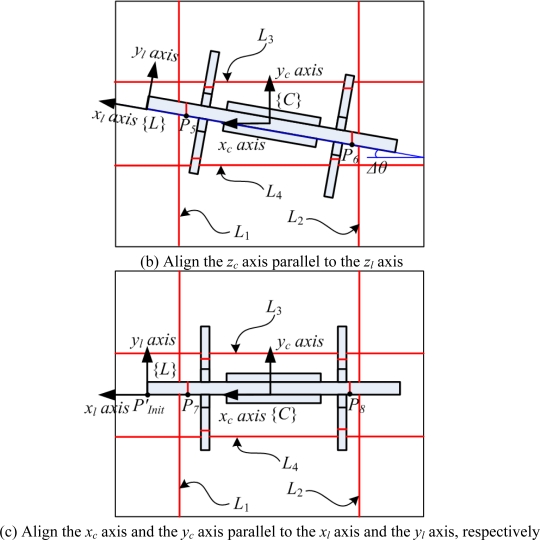
Top view alignment with four laser lines, *L*_1_, *L*_2_, *L*_3_ and *L*_4_, projected by the laser diodes, *D*_1_, *D*_2_, *D*_3_ and *D*_4_, respectively.

Through the top view alignment, the rough lug frame is obtained as shown in Figure 9. In Figure 9a, the *z_l_* axis of {*L*} can be obtained by the surface normal to the steel plate as follows:

(6) $${\vec{z}}_{l}=\vec{n}=\vec{P_{1}P_{2}}\times \vec{P_{1}P_{3}}$$

where *z⃗_l_* is a unit vector along the *z_l_* axis, and *P_i_* for *i*=1,2,3,4 are intersections of each pair of lines. In this case, the intersection *P_i_*(*x_i_*,*y_i_*,*z_i_*) can be easily obtained by using its mapped point *p_i_*(*x′_i_*,*y′_i_*) onto the image plane and Equation 1.

For obtaining *p_i_* from the camera image, we first separate the projected laser lines from the background using the vertical threshold algorithm [9] robust to the illumination change. Next, the Zhang-Suen thinning algorithm [10] is applied to the threshold image. And then, the Hough transform algorithm [11] is applied to the thinning image for obtaining each laser line equation in *x′* and *y′* as follows:

(7) $$\rho_{i}=x^{\prime }\;\text{cos}\;\theta_{i}+y^{\prime }\;\text{sin}\;\theta_{i}$$

where *ρ_i_* is the distance from the origin of the image plane to the laser line *L_i_*, and *θ_i_* is the angle between the normal line to *L_i_* and the *x′* axis. Thus, the point *p*_1_ can be obtained by solving the following linear system of *L*_1_ and *L*_3_.

(8) $$\left[\begin{array}{cc} {\text{cos}\;\theta }_{1} & {\text{sin}\;\theta }_{1}\\ {\text{cos}\;\theta }_{3} & {\text{sin}\;\theta }_{3} \end{array}\right]\;\left[\begin{array}{c} x^{\prime }\\ y^{\prime } \end{array}\right]=\left[\begin{array}{c} \rho_{1}\\ \rho_{3} \end{array}\right]$$

Similarly, the points, *p*_2_, *p*_3_ and *p*_4_, can be obtained. Then, the robot is controlled to align the *x_c_* axis of {*C*} with the obtained *x_l_* axis of {*L*} in parallel.

After the *z_c_* axis alignment with the *z_l_* axis, the camera image is obtained as shown in Figure 9b. Here, the robot is controlled to align the *x_c_* axis with the *x_l_* axis. In this case, the *x_l_* axis is parallel to the vector $\vec{P_{6}P_{5}}$ from *P*_6_ to *P*_5_, where *P*_5_ and *P*_6_ are the points on the laser lines, *L*_1_ and *L*_2_, projected on the central beam of the lug, respectively. Thus, the difference angle *Δθ* between the *x_c_* axis and the *x_l_* axis can be obtained as follows:

(9) $$\Delta \theta ={\text{cos}}^{-1}\left(\frac{\vec{P_{6}P_{5}}•\vec{x_{l}}}{\left\|\vec{P_{6}P_{5}}\right\|}\right)$$

where *x⃗_l_* is a unit vector along the *x_l_* axis. By the robot rotation of *Δθ*, the *x_c_* axis can be aligned with the *x_l_* axis. As a result of the *x_c_* alignment with the *x_l_* axis, the *y_c_* axis is also aligned with the *y_l_* axis.

**Figure 10.**
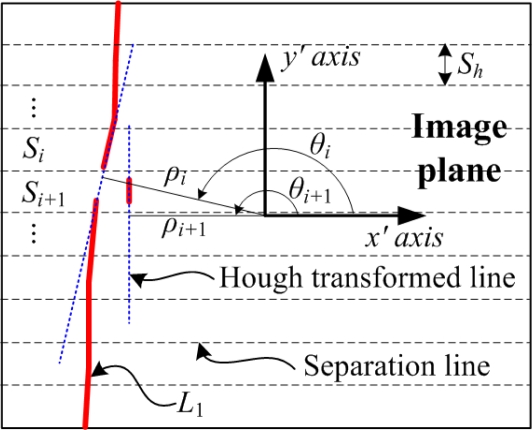
Separated Hough transform for obtaining the line segment of *L*_1_ projected on the lug center.

In Figure 9b, the points, *P*_5_ and *P*_6_, are obtained as follows. First, the line segments on the central beam are separated from the segments on the background by the separated Hough transform algorithm proposed in this study. This algorithm separates the image into several sections at the interval of *S_h_*, and then applies the Hough transform to each section *S_i_* for *I* = 1,2,…,*N* as shown in Figure 10. As a result of the separated Hough transform, the line parameters, *ρ_i_* and *θ_i_*, for the line segment in *S_i_* can be obtained by the maximum voting parameters. Next, two line segments in the consecutive sections, *S_i_* and *S*_*i*+1_, are merged as one segment if |*ρ_i+_*_1_−*ρ_i_*| < *ε*_1_ and |*θ_i+_*_1_−*θ_i_*| < *ε*_2_ are satisfied, where *ε*_1_ and *ε*_2_ are the acceptable boundaries for the same line. The line segment merging is repeated until there is no line segment satisfying the same line conditions. As a result, the line segment projected on the central beam can be obtained since its line parameters are clearly distinguished from those of the background line segments. Finally, from two central beam line segments, two points, *P*_5_ and *P*_6_, can be obtained by Equation 1.

After each axis alignment of {*C*} with {*L*}, the camera image is obtained as shown in Figure 9c. By the separated Hough transform, the points, *P*_7_ and *P*_8_, on the central beam can be obtained similarly. In this case, the initial point *P′_Init_* of the lug is obtained by using the lookup table (LUT) about the central beam shape of the lug, where the LUT is manually formed by measuring the height of the lug along the *z_l_* axis at regular intervals along the *x_l_* axis. In this case, since the *z_c_* axis is aligned with the *z_l_* axis, the height of the lug at *P*_7_ can be calculated by the camera as the difference between the depth of the steel plate and that of *P*_7_ along *z_c_* axis. Then, the *x_l_* position for the lug height at *P*_7_ can be obtained by using the LUT. The absolute value of the *x_l_* position is same as the distance *d* between *P′_Init_* and *P*_7_ along the *x_l_* axis (or *x_c_* axis). Using the position of *P*_7_ relative to {*C*} and the distance *d* between *P*_7_ and *P′_Init_*, the point *P′_Init_* can be obtained relative to {*C*}. However, the obtained lug frame {*L*} is not precise enough to carry out automatic robot welding as mentioned in Section 2.2. Therefore, the additional fine alignment is needed.

### 3.2. Precise Welding Line Detection

**Figure 11.**
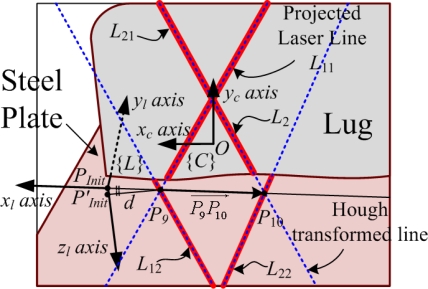
Side view alignment with two laser lines, *L*_1_ and *L*_2_, projected by the laser diodes, *D*_1_ and *D*_2_, respectively.

For successful automatic robot welding, the precise welding line detection is very critical. Thus, the robot is controlled to move close to the side of the lug, and then the side view alignment is carried out with two laser lines *L*_1_ and *L*_2_ as shown in Figure 11. In this case, *L*_11_ and *L*_12_ represent the laser line segments of *L*_1_ projected onto the side of the lug and the steel plate, respectively. By the same way, *L*_21_ and *L*_22_ represent the laser line segments of *L*_2_ projected onto the lug side and the plate, respectively. Here, the line equations of *L*_11_, *L*_12_, *L*_21_ and *L*_22_ can be obtained by the threshold, thinning, and Hough transform algorithms like Equation 7 in Section 3.1. Then, the intersection *p*_9_(*x′*_9_,*y′*_9_) between *L*_11_ and *L*_12_ and the intersection *p*_10_(*x′*_10_,*y′*_10_) between *L*_21_ and *L*_22_ are obtained like Equation 8. From the points, *p*_9_ and *p*_10_, on the image plane, the real points, *P*_9_ and *P*_10_, can be obtained by Equation 1. In this case, the parametric line equation of the welding line is obtained by two points, *P*_9_ and *P*_10_, as follows:

(10) $$\vec{{\mathrm{OP}}_{w}}=\vec{{\mathrm{OP}}_{9}}+t\vec{P_{9}P_{10}}$$

where $\vec{{\mathrm{OP}}_{w}}$, $\vec{{\mathrm{OP}}_{9}}$ and $\vec{{\mathrm{OP}}_{10}}$ are the position vectors on the welding line from the origin of {*C*}, $\vec{P_{9}P_{10}}$ is ( $\vec{{\mathrm{OP}}_{10}}-\vec{{\mathrm{OP}}_{9}}$), and *t* is the parameter in (−∞,∞). For automatic robot welding, the robot is controlled to follow the welding line from the initial point. However, the initial point *P′_Init_* obtained by the top view alignment may not locate on the welding line because of its position error. In this case, the robot cannot continuously follow the welding line from *P′_Init_* because of the discontinuity between the welding line and *P′_Init_*. As a result, there is little possibility of robot welding successfully. Thus, for removing the discontinuity, we newly define the initial point *P_Init_* to locate on the welding line at a distance of *d* from *P*_9_ as shown in Figure 11. The new initial point *P_Init_* can be obtained as follows:

(11) $$\vec{{\mathrm{OP}}_{\mathrm{Init}}}=\vec{{\mathrm{OP}}_{9}}-d\frac{\vec{P_{9}P_{10}}}{\left\|\vec{P_{9}P_{10}}\right\|}$$

This way, the precise initial point and the welding line equation can be obtained by the proposed sensor.

## 4. Experimental Results and Discussion

**Figure 12.**
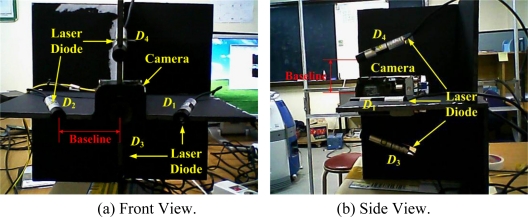
Prototype of the proposed 3D lug pose detection sensor with a monocular camera and four laser line diodes.

Experiments for the top view alignment and the side view alignment were sequentially carried out with a prototype of the 3D lug pose detection sensor as shown in Figure 12. The design parameters of the sensor were determined, taking the detectable range and the sensor resolution into consideration, as shown in Table 2.

**Figure 13.**
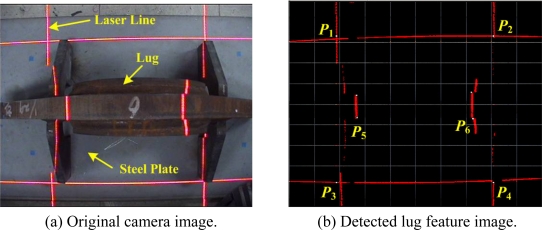
Results of feature extraction from the top view image.

First, we carried out the top view alignment at a distance of about 71.0 cm to the lug, as shown in Figure 13. By successively applying the vertical threshold, thinning, Hough transform and separated Hough transform algorithms to the original image in Figure 13a, the feature points, *P*_1_, *P*_2_, *P*_3_ and *P*_4_, on the steel plate were obtained as shown in Figure 13b. From the obtained feature points, the surface normal *n⃗* to the steel plate could be calculated, where *n⃗* = *z⃗_l_*. As a result, the angular error between the normal vector *n⃗* and the actually measured vector was just 0.03°. Thus, the robot can be controlled to align the *z_c_* axis with the *z_l_* axis. Then, the feature points, *P*_5_ and *P*_6_, on the central beam of the lug were obtained to align the *x_c_* axis with the *x_l_* axis. Using the feature points, the angle between the *x_c_* axis with the *x_l_* axis could be obtained as *Δθ* = 0.05°. The robot can be controlled to align the *x_c_* axis with the *x_l_* axis again. Finally, the rough initial point *P′_Init_* could be obtained by the lookup table (LUT), which mapped the *x_l_* position of the lug to the *z_l_* position relative to {*L*} as shown in Table 3. Here, for each *x_l_* position of the lug, the *z_l_* position was manually measured in advance.

**Figure 14.**
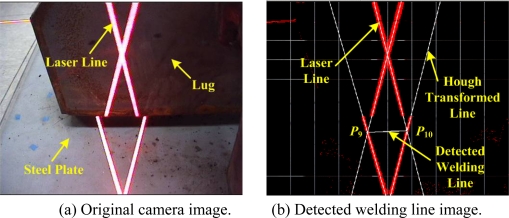
Results of the feature extraction from the side view image.

In accordance with the rough lug frame, the robot can be controlled to move close to the side of the lug. Next, we carried out the side view alignment at a distance of about 25.5 cm to the lug as shown in Figure 14. Similarly to the top view alignment, from the original image in Figure 14a, the feature points, *P*_9_ and *P*_10_, were obtained as shown in Figure 14b. Finally, the precise initial position *P_Init_* on the welding line could be obtained by the distance from *P*_9_ to *P′_Init_* from the LUT.

**Figure 15.**
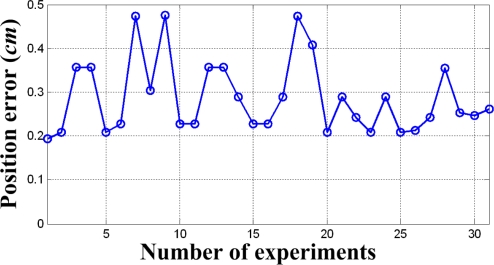
Position error of the initial welding position versus the number of experiments.

As a result of 31 times experiments, the errors of the initial welding position *P_Init_* were obtained as shown in Figure 15. In this case, the proposed sensor had the average error of 0.29 cm, the standard deviation of 0.0844 cm, the maximum error of 0.48 cm, and the minimum error of 0.19 cm. In this case, 68% of the 31 position errors are less than 0.3 cm, and 87% of them are less than 0.4 cm. The position error of the proposed sensor system is small enough to practically weld the lug in the field of shipbuilding.

## 5. Conclusions

A precise 3D lug pose detection sensor which consists of a camera and four laser line diodes was proposed for automatic robot welding of the lug to the huge steel plate. The lug pose, consisting of position and orientation, could be obtained from the coarse-to-fine alignment. In this case, the vertical threshold, thinning, Hough transform and separated Hough transform algorithms, which are robust to illumination change, were used for robustly extracting feature points from the camera image. As a result of the coarse-to-fine alignment with the proposed sensor, the lug pose could be obtained precise enough to automatically weld the lug to the steel plate. In the experiments, the initial position on the welding line could be obtained with the average error of 0.29 cm, the standard deviation of 0.0844 cm, the maximum error of 0.48 cm, and the minimum error of 0.19 cm. The experimental results are acceptable for practical lug welding in the field of shipbuilding. Consequently, the proposed sensor is expected to make technological innovations of productivity and quality for shipbuilding automation.

## Figures and Tables

**Table 1.** Automatic robot welding procedure.

Procedure: automatic robot welding
**[Step1] Top view alignment (Coarse alignment)**-From the top view image of the lug, obtain the rough lug frame with the lookup table (LUT) about the lug shape, and align the camera frame with the obtained lug frame as follows: Obtain the top view image of the lug. Extract the feature points of the projected laser lines on the lug. Compute the lug frame with the obtained feature points and the LUT. Align the camera frame {*C*} with the lug frame {*L*}. Move the robot close to the left side of the lug. **[Step 2] Side view alignment (Fine alignment)**-From the side view image of the lug, obtain the exact welding line, and then estimate the initial welding position of the lug as follows: Obtain the side view image of the lug. Extract the feature points of the projected laser lines on the lug. Compute the welding line equation from the obtained feature points. Estimate the initial welding position of the lug with the welding line equation. Move the robot to the initial welding position for automatic welding. **[Step 3] Automatic robot welding**-Control the robot to weld the lug according to the predefined welding path.

**Table 2.** Sensor parameters used for experiments.

**Sensor Parameters**	**Descriptions**
*b* = 7 cm	Baseline between the camera and each laser line diode
*α* = 70°	Projection angle of diodes, *L*_1_ and *L*_2_
*β* = 96.5°	Projection angle of diodes, *L*_3_ and *L*_4_
*ε*_1_ = 5 pixels	Acceptable boundary of the line parameter *ρ* for the same line
*ε*_2_ = 5°	Acceptable boundary of the line parameter *θ* for the same line

**Table 3.** Lookup table (LUT) about the central beam shape of the lug (unit cm).

***x****_l_*	**0**	**−1**	**−2**	**−3**	**−4**	**−5**	**−6**	**−7**	**−8**	**−9**	**−10**
***z****_l_*	−10.3	−11.3	−12.4	−13.4	−14.4	−15.5	−16.5	−17.6	−18.6	−19.7	−20.7
***x****_l_*	**−12**	**−13**	**−14**	**−15**	**−16**	**−17**	**−18**	**−19**	**−20**	**−21**	**−22**
***z****_l_*	−22.8	−23.8	−24.8	−25.9	−26.9	−27.9	−28.9	−30.0	−31.0	−31.3	−31.5
***x****_l_*	**−24**	**−25**	**−26**	**−27**	**−28**	**−29**	**−30**	**−31**	**…**		
***z****_l_*	−32.0	−32.3	−32.5	−32.8	−33.0	−33.3	−33.5	−33.5	…		
